# The complete chloroplast genome characteristics of *Polygala crotalarioides* Buch.-Ham. ex DC. (*Polygalaceae*) from Yunnan, China

**DOI:** 10.1080/23802359.2021.1964396

**Published:** 2021-09-06

**Authors:** Jiayu Ma, Junmin Wang, Congying Li, Jie Liu, Can Chen, Yan Hua

**Affiliations:** a Key Laboratory for Forest Resources Conservation and Utilisation in the Southwest Mountains of China Southwest Forestry University, Ministry of Education, Kunming, China; b Yunnan Yihua Biological Co., Ltd, Shuangjiang, China; c Key Laboratory of Dairy Science, Ministry of Education, Northeast Agricultural University, Harbin, China

**Keywords:** Chloroplast genome, phylogenetic analysis, *Polygala crotalarioides*

## Abstract

*Polygala crotalarioides* Buch.-Ham. ex DC. (Polygalaceae) is a perennial herbaceous plant widely distributed in southwest China. Its chloroplast genome sequence was obtained using Illumina NovaSeq sequencing technology, and then was assembled, annotated, and characterized. The total length of chloroplast genome is 164,269 bp and it contained a large (LCS, 82,697 bp) and a small (SSC, 8083 bp) single copy region, separated by a pair of inverted repeats (IRA/IRB, 36,744 bp). The overall GC content of genome was 36.8% and corresponding values of the LSC, SSC, and IR regions are 34.9%, 29.5%, and 39.7%, respectively. There are 135 genes that were predicted including 89 protein-coding, 38 tRNA, and 8 rRNA genes. The maximum-likelihood (ML) phylogenetic tree reconstructed by IQ-TREE indicated that *P. crotalarioides* had a strong genetic relationship with *P. tenuifolia*, *P. sibirica*, and *P. japonica*. In summary, the complete chloroplast genome of *P. crotalarioides* is reported for the first time, which is of great significance for future research on the evolutionary phylogeny and protection of *Polygala*.

*Polygala crotalarioides* is a perennial herb classified in the Polygalaceae. It is distributed in southwest and southern China and is an endangered species (Zhang et al. 2002). *Polygala crotalarioides* is considered a rare Chinese herbal medicine used by the Wa people from the Yunnan Province. This plant has excellent biological activities and is widely used in folk to fight fatigue (Xiang and Zhang 1995), calm the mind, and tonic the heart. Research shows that *P. crotalarioides* has anti-oxidative and anti-fatigue effects and improves the body’s ability to adapt to stress (Liu and Ma 2014). Analysis of the chloroplast genome will contribute to the bioinformatics and evolutionary history of this species.

Chloroplast genomes can provide reliable data to determine genetic and evolutionary relationships in plants (Kugita et al. 2003). So far, complete chloroplast genomes of several species from the Polygalaceae have been studied and deposited in GenBank, including *P. japonica* (Zuo et al. 2021). At present, the chloroplast genome of *P. crotalarioides* has not been deciphered. In this paper, we assembled and annotated the chloroplast genome of *P. crotalarioides* using high-throughput sequencing. These data provide a reference for further molecular research on the taxonomy and evolutionary systematic of the Polygalaceae.

Fresh leaves of *P. crotalarioides* were collected from Shuangjiang County (Yunnan, China; geospatial coordinates: 99.844°E, 23.514°N; Altitude: 1015.9 m). The total genomic DNA was extracted using the Magnetic beads plant genomic DNA preps Kit (Annoroad Biological Technology, Yiwu, China). The herbarium specimen was deposited at the Herbarium of Southwest Forestry University Southwest Forestry University, Kunming, China (http://bbg.swfu.edu.cn/, Shuang-Zhi Li, dawn723@163.com) under the voucher number PC20200910-5.

The Illumina NovaSeq 6000 platform was used for sequencing and the read length was 150 bp paired end. The filtered chloroplast reads were assembled from scratch using GetOrganelle. Then, we selected *P. tenuifolia* as the reference sequence, using Geneious R8 (Biomatters Ltd, Auckland, New Zealand), assembled, and performed the annotation of the complete chloroplast genome. Finally, the chloroplast DNA sequence and complete annotation were deposited in GenBank under accession number MW543308.1.

The chloroplast genome is 164,268 bp in length and consists of an LSC (82,697 bp) and SSC (8,083 bp), separated by two inverted repeats (36,744 bp). The total GC content in the LSC (34.9%) and SSC (29.5%) was 36.8%, which was lower than that in the IR regions (39.7%). The circular genome contains 135 genes, including 89 protein-coding (PCG), 38 tRNA, and 8 rRNA genes. Among the 135 annotated genes, there are 84 single copy genes and 25 duplicate genes. The protein-coding genes, tRNA and rRNA of *P. crotalarioides* chloroplast genome are identical in quantity with *P. japonica*, but they are different in total length, total GC content, etc., which may be caused by differences in species and gene richness (Zuo et al. 2021).

To uncover the phylogenetic relationship of *P. crotalarioides* in Polygalaceae family, the chloroplast genome sequences of 14 plants including *P. crotalarioides* were selected to construct the phylogenetic trees. The alignment of all cp genomes was done by the MAFFT version 7 software (Katoh and Standley 2013), which was analyzed by IQ-TREE 1.5.5 (Nguyen et al. 2015) under the TVM + F+R2 nucleotide substitution model (Kalyaanamoorthy et al. 2017). The phylogenetic analysis 1000 bootstrapping replicates was performed based on the maximum likelihood (ML) tree. Phylogenetic analysis showed that *Polygala crotalarioides* was strongly related to *P. tenuifolia*, *P. sibirica*, and *P. japonica* with 100% bootstrap support (Figure 1). Among them, the chloroplast structure of *P. crotalarioides* we studied was highly consistent with that of *P. tenuifolia* of the same genus previously published (Lee et al. 2020). In conclusion, the complete chloroplast genome of *P. crotalarioides* provides useful DNA data for further biological analysis and the evolutionary phylogenetics of the Polygalaceae.

**Figure 1.**
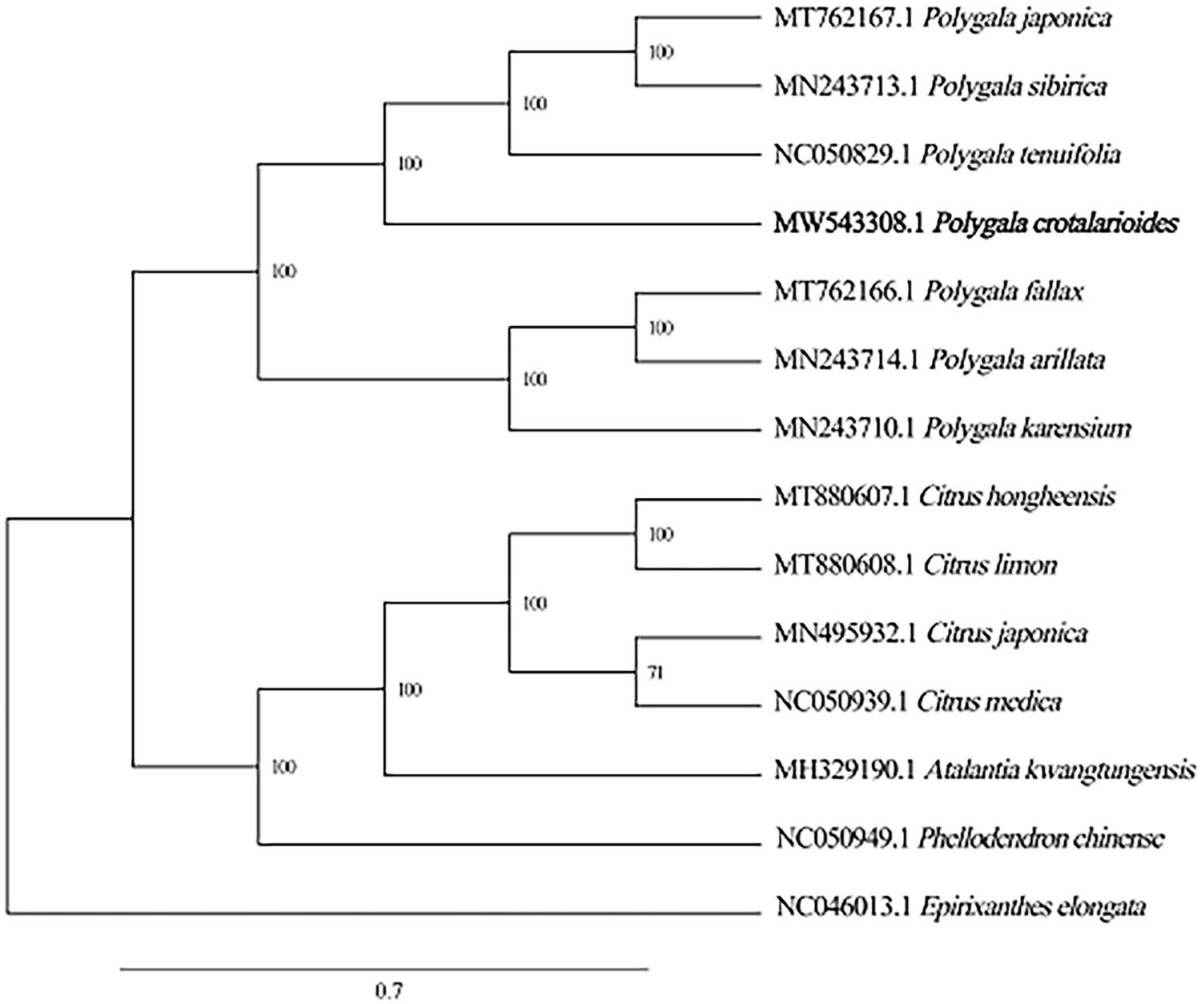
Neighbor-Joining tree of *P. crotalarioides* and related species using chloroplast sequences.

## Data Availability

The genome sequence data that support the findings of this study are openly available in GenBank of NCBI at [https://www.ncbi.nlm.nih.gov] under the accession number MW543308.1. The associated BioProject, SRA, and Bio-Sample numbers are PRJNA732229, SRR14679146, and SAMN19314347 respectively.
